# Hyperosmia and Hypergeusia As Potential Clues in Diagnosing Adrenal Insufficiency: A Case Report

**DOI:** 10.7759/cureus.59038

**Published:** 2024-04-25

**Authors:** Senna Shimazaki, Etaro Hashimoto, Haruka Kuno, Takami Maeno, Nako Matsumoto, Masatsune Suzuki, Tetsuhiro Maeno

**Affiliations:** 1 Department of General Medicine and Primary Care, University of Tsukuba Hospital, Tsukuba, JPN; 2 Department of General Medicine, Mito Kyodo General Hospital, Mito, JPN; 3 Department of Primary Care and Medical Education, Faculty of Medicine, University of Tsukuba, Tsukuba, JPN; 4 Department of Endocrinology and Metabolism, University of Tsukuba Hospital, Tsukuba, JPN

**Keywords:** history taking, anorexia, adrenal insufficiency, hypergeusia, hyperosmia

## Abstract

Adrenal insufficiency often presents with nonspecific symptoms, physical findings, and laboratory results, leading to diagnostic challenges. However, reports have indicated that specific symptoms such as hypergeusia (hypersensitivity to taste) and hyperosmia (hypersensitivity to smell) can also occur. We report the case of a 60-year-old male with loss of appetite, fatigue, and polyarthralgia, where a detailed medical history revealed the cause of anorexia to be hypergeusia and hyperosmia. These specific symptoms led to the diagnosis of adrenal insufficiency. Treatment with oral steroids for secondary adrenal insufficiency resulted in the improvement of his diverse symptoms. This case illustrates that in patients presenting with chronic nonspecific symptoms, inquiring about heightened taste and smell sensitivity can prompt suspicion of adrenal insufficiency. Moreover, this case serves as a reminder that careful medical history taking in patients with nonspecific symptoms can uncover specific findings that may be diagnostic clues.

## Introduction

Adrenal insufficiency is often characterized by nonspecific symptoms and lacks specific findings on physical examination and routine laboratory tests, making diagnosis difficult [1]. On the other hand, reports have suggested that many, if not most, patients with adrenal insufficiency may exhibit hypergeusia and hyperosmia [2,3]. We experienced a case where a detailed inquiry into nonspecific symptoms, such as loss of appetite, led to the identification of more specific signs, hypergeusia and hyperosmia, resulting in the diagnosis of adrenal insufficiency. This report emphasizes the importance of evaluating hypergeusia and hyperosmia in the diagnosis of adrenal insufficiency and highlights the significance of meticulous medical history taking to identify specific symptoms from nonspecific clinical presentations.

## Case presentation

The patient, a 60-year-old Japanese male with a history of hyperuricemia and no prior steroid use, presented with a three-month history of anorexia, fatigue, and joint pain in both shoulders, elbows, and fingers. Accompanied by the loss of appetite, he experienced a weight loss of 9 kg, from 60 kg to 51 kg, over three months. Despite undergoing blood tests, urine tests, upper gastrointestinal endoscopy, and whole-body PET-CT at another hospital, no abnormalities were noted. He was referred to our institution for further investigation. The primary complaint was nonspecific: loss of appetite. To elucidate the specific cause of his anorexia, a systematic inquiry was conducted using close-ended questions about changes in taste and smell, dry mouth, dysphagia, early satiety, and postprandial gastrointestinal symptoms (nausea, vomiting, abdominal pain). As a result, it became evident that the three symptoms of hypergeusia, hyperosmia, and dry mouth were the causes of his anorexia. He experienced a heightened sensitivity to food odors and a salty taste, leading to decreased appetite. He also became unable to eat dry solid foods like bread without liquids, resulting in a diet that consisted mainly of jelly, porridge, and cereal with milk. No dry eyes were reported. His wife had prepared his meals without changing ingredients or seasoning. He had a history of hyperuricemia managed only with dietary advice, and without any medication. He did not use over-the-counter drugs or supplements. His mother had rheumatoid arthritis. He had a history of consuming 700 mL of whiskey soda daily but had stopped drinking since the onset of symptoms. Similarly, he had quit smoking, previously having smoked 10 cigarettes a day. His usual blood pressure was around 130/90 mm Hg, but at the time of examination, it was low at 86/61 mm Hg. His pulse was 92 beats per minute, oxygen saturation (SpO_2_) was 98% on ambient air, and body temperature was 36.8°C. He exhibited tenderness in the joints of both shoulders, elbows, hands, and fingers, but no swelling, heat, or redness. No skin pigmentation was observed. Other physical examinations were unremarkable. Blood tests showed mild normocytic anemia (Hb 11.5 g/dL, mean corpuscular volume 89.4 fL) and mild eosinophilia (white blood cell count 8000 /μL, eosinophils 544 /μL), with no other abnormalities, including thyroid function (Table 1).

**Table 1:** Initial blood test results

Component	Lab value	Reference range and units
White blood cell count	8.0	4.0 - 9.0 ×10³/μL
Segmented cell	46.3	45 - 55 %
Lymphocyte	41.5	25 - 45 %
Monocyte	5.0	4.0 - 7.0 %
Eosinophil	6.8	1.0 - 5.0 %
Basophil	0.4	0 - 1.0 %
Eosinophil count	544	0-500 /μL
Red blood cell count	3.98	4.27 - 5.7 ×10⁶/μL
Hemoglobin	11.5	14 - 18 g/dL
Hematocrit	35.6	40 - 52 %
Mean corpuscular volume	89.4	80 - 100 fL
Platelet count	219	150 - 350 ×10³/μL
Total protein	6.0	6.7 - 8.3g/dL
Albumin	4.0	3.8 - 5.3 g/dL
Aspartate aminotransferase	29	8.0 - 38 U/L
Alanine aminotransferase	24	4.0 - 44 U/L
Lactate dehydrogenase	173	124 - 222 U/L
Creatinine	0.92	0.61 - 1.04 mg/dL
Blood urea nitrogen	14.9	8.0 - 20 mg/dL
Sodium	141	135 - 147 mEq/L
Potassium	4.1	3.6 - 5.0 mEq/L
Chloride	101	98 - 108 mEq/L
C-reactive protein	0.25	0 - 0.2 mg/dL
Erythrocyte sedimentation rate	19	2.0 - 10 mm/h
Fasting blood glucose	85	70 - 109 mg/dL
Hemoglobin A1c	5.4	4.6 - 6.2 %
Free thyroxine	1.19	0.9 - 1.7 ng/dL
Thyroid-stimulating hormone	2.78	0.5 - 5.0 μIU/mL
anti-SSa antibody	<1.0	0 - 9.9 U/mL
anti-SSb antibody	1.0	0 - 9.9 U/mL
Cortisol	0.7	6.4 - 21 ㎍/dL
Adrenocorticotropic hormone	5.7	7.2 - 63.3 pg/mL

Given the chronic course of his diverse systemic symptoms and the specific signs of taste and smell hypersensitivity, adrenal insufficiency was strongly suspected. Early morning fasting blood tests showed cortisol levels of 0.7 μg/dL (normal range 6.4 - 21 μg/dL) and adrenocorticotropic hormone (ACTH) levels of 5.7 pg/mL (normal range 7.2 - 63.3 pg/mL), both of which were low, leading to the diagnosis of adrenal insufficiency. Chronic joint pain and dry mouth prompted differential diagnoses of Sjögren's syndrome and rheumatoid arthritis, but antibody tests were normal. The dry mouth was thought to be symptomatic of dehydration associated with anorexia. Subsequently, the patient was referred to the endocrinology department, where an evaluation suggested hypopituitarism with a predominant impairment in ACTH secretion. Treatment with 15 mg/day of hydrocortisone for secondary adrenal insufficiency was initiated. A few days after starting treatment, the hypersensitivity to smells and tastes improved, and his appetite returned. Subsequently, the dry mouth and fatigue improved, and his blood pressure returned to normal levels. His weight increased by 2 kg two months after starting treatment.

## Discussion

This case highlighted the significance of hyperosmia and hypergeusia as key indicators in diagnosing adrenal insufficiency [2,3]. Adrenal insufficiency can result from primary adrenal damage due to autoantibodies or infections, secondary adrenal insufficiency due to a deficiency in ACTH, or suppression of ACTH due to exogenous glucocorticoids or opioids. It is characterized by nonspecific symptoms such as anorexia (53-67%), weight loss (66-76%), fatigue (84-95%), and joint and muscle pain (36-40%) [1,4]. Hypotension is common, and apart from pigmentation of the skin and oral mucosa seen in primary adrenal insufficiency, there are no specific physical findings. Laboratory findings may include normocytic anemia (11-15%), eosinophilia (32%), hyponatremia (70-80%), and hypoglycemia (20%) [4,5]. Given the nonspecific nature of the symptoms, physical findings, and laboratory results, diagnosing adrenal insufficiency is often challenging [1]. However, impairments in taste and smell are also recognized manifestations of adrenal insufficiency [6], and there have been poorly recognized reports where all patients with adrenal insufficiency displayed hypergeusia and hyperosmia [2,3]. Although the actual prevalence of these symptoms remains uncertain, the meticulous medical history taking that identified hypergeusia and hyperosmia provided crucial diagnostic clues in this case. Early diagnosis may be possible in patients with chronic nonspecific symptoms by considering adrenal insufficiency in the differential diagnosis and specifically inquiring about hypergeusia and hyperosmia. The mechanism behind hypergeusia and hyperosmia in adrenal insufficiency patients remains unclear, but understanding this pathology may allow for earlier detection of sensory changes.

While hypergeusia and hyperosmia are rare symptoms, it is crucial not to overlook them as causes of anorexia. As patients may not spontaneously report these hypersensitivities, as seen in this case, systematic questioning in patients presenting with anorexia should include inquiries about hypergeusia and hyperosmia using close-ended questions. This approach also teaches us that even with nonspecific symptoms like anorexia, meticulous medical history-taking can uncover specific symptoms. This finding underscores the importance of hypothesis generation by physicians when observing a clinical phenomenon [7].

## Conclusions

Adrenal insufficiency often presents with nonspecific symptoms, making diagnosis challenging. However, hyperosmia and hypergeusia may represent more specific symptoms in patients with adrenal insufficiency. In patients with chronic nonspecific symptoms, including anorexia, a detailed medical history and inquiry into the presence of hypergeusia and hyperosmia can serve as potential clues in diagnosing adrenal insufficiency.
